# Supplementary material for: Functional Characterization of Mouse and Human Arachidonic Acid Lipoxygenase 15B (ALOX15B) Orthologs and of Their Mutants Exhibiting Humanized and Murinized Reaction Specificities
Source: Int J Mol Sci. 2023 Jun 12;24(12):10046. doi: 10.3390/ijms241210046 (PMC10298594; doi:10.3390/ijms241210046)
Supplement: Supplementary file 1 [file ijms-24-10046-s001.zip › ijms-2317160-supplementary.pdf]

Supplemental Material to the Paper

Functional Characterization of Mouse and Human Arachidonic Acid Lipoxygenase 15B (ALOX15B) Orthologs and of Their Mutants Exhibiting Humanized and Murinized Reaction Specificities

Kumar R. Kakularam [1,†], Miquel Canyelles-Niño [2,3,†], Xin Chen [1], José M. Lluch [2,4], Àngels González-Lafont [2,4] and Hartmut Kuhn [1,*]

[1] Department of Biochemistry, Charité–Universitätsmedizin Berlin, Corporate Member of Freie Universität Berlin and Humboldt Universität zu Berlin, D-10117 Berlin, Germany; kumar.1416@gmail.com (K.R.K.); xin.chen2@charite.de (X.C.)

[2] Departament de Química, Universitat Autònoma de Barcelona, 08193 Barcelona, Spain; miquel.canyelles@uab.cat (M.C.-N.); josemaria.lluch@uab.cat (J.M.L.); angels.gonzalez@uab.cat (À.G.-L.)

[3] Arquebio S.L., 08005 Barcelona, Spain

[4] Institut de Biotecnologia i de Biomedicina (IBB), Universitat Autònoma de Barcelona, 08193 Barcelona, Spain

* Correspondence: hartmut.kuehn@charite.de; Tel.: +49-30-450528040

† These authors contributed equally to this work.

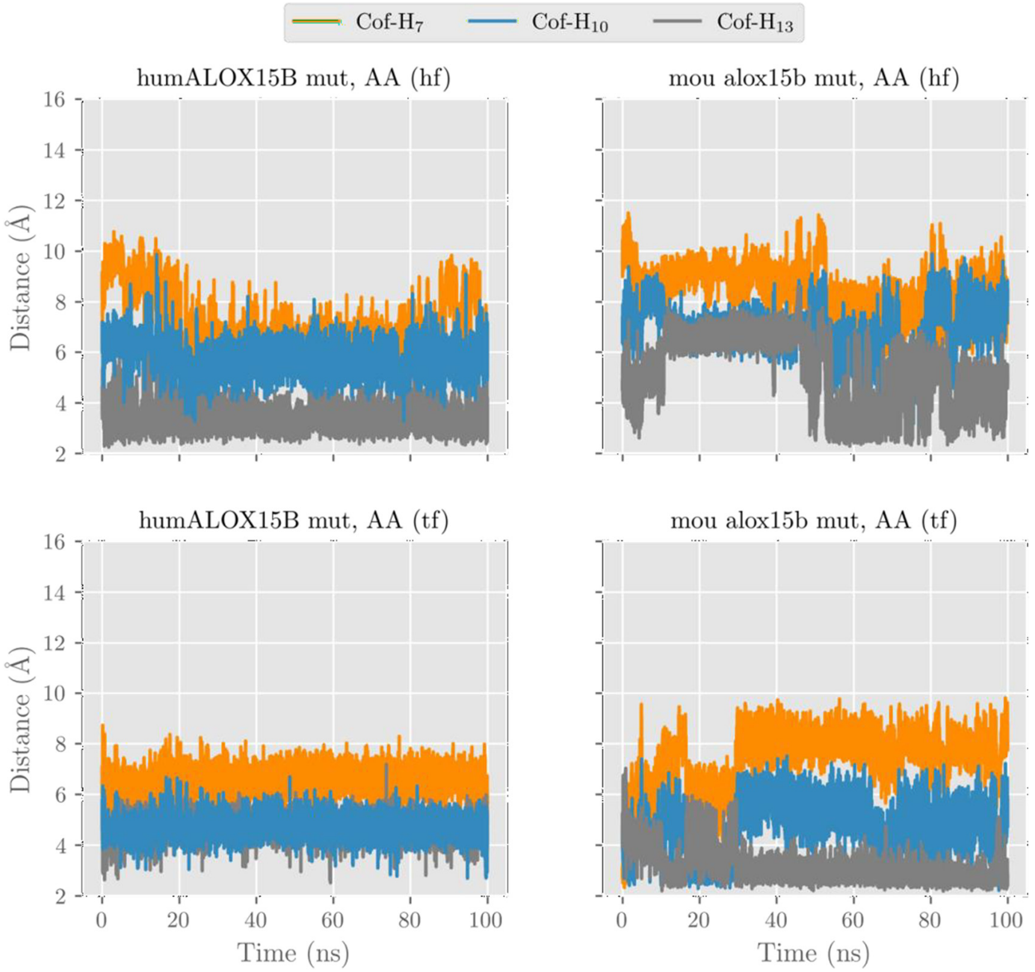

Figure S1. Molecular dynamics simulations of the arachidonic acid complexes of human and mouse ALOX15B mutants. For these simulations we first worked out 3D models for the Asp602Tyr+Val603His double mutant of human ALOX15B and the Tyr603Asp+His604Val double mutant of mouse alox15b. Then arachidonic acid (AA) was docked into the substrate binding pocket of the enzymes in its head-first (hf) and its tail-first (tf) orientations, and the distances from the oxygen atom in the Fe(III)-OH^- cofactor to the closest hydrogen atom attached to the three pro-chiral bisallylic methylenes C7 (H7), C10 (H10) and C13 (H13) along the Molecular Dynamics simulations are plotted. Head-first (hf) AA/humALOX15B double mutant (upper left) shows a stable position, being the H13 the closest hydrogen, so the one with the most expected reactivity. For the same system but with the AA in a tail-first (tf) conformation (lower left) the results are similar, but H10 and H13 are both in similar distance to the cofactor. For the mouse Alox15b double mutant, the AA in head-first (hf) orientation (upper right) reaches a reactive conformation after 50 ns. H13 is the closest to the cofactor. Nonetheless, the overall stability of the substrate in the cavity is not as good as in the other systems. On the contrary, when AA is in tail-first conformation (tf, lower right), the position of AA is more stable and H13 are in closest proximity to the cofactor.

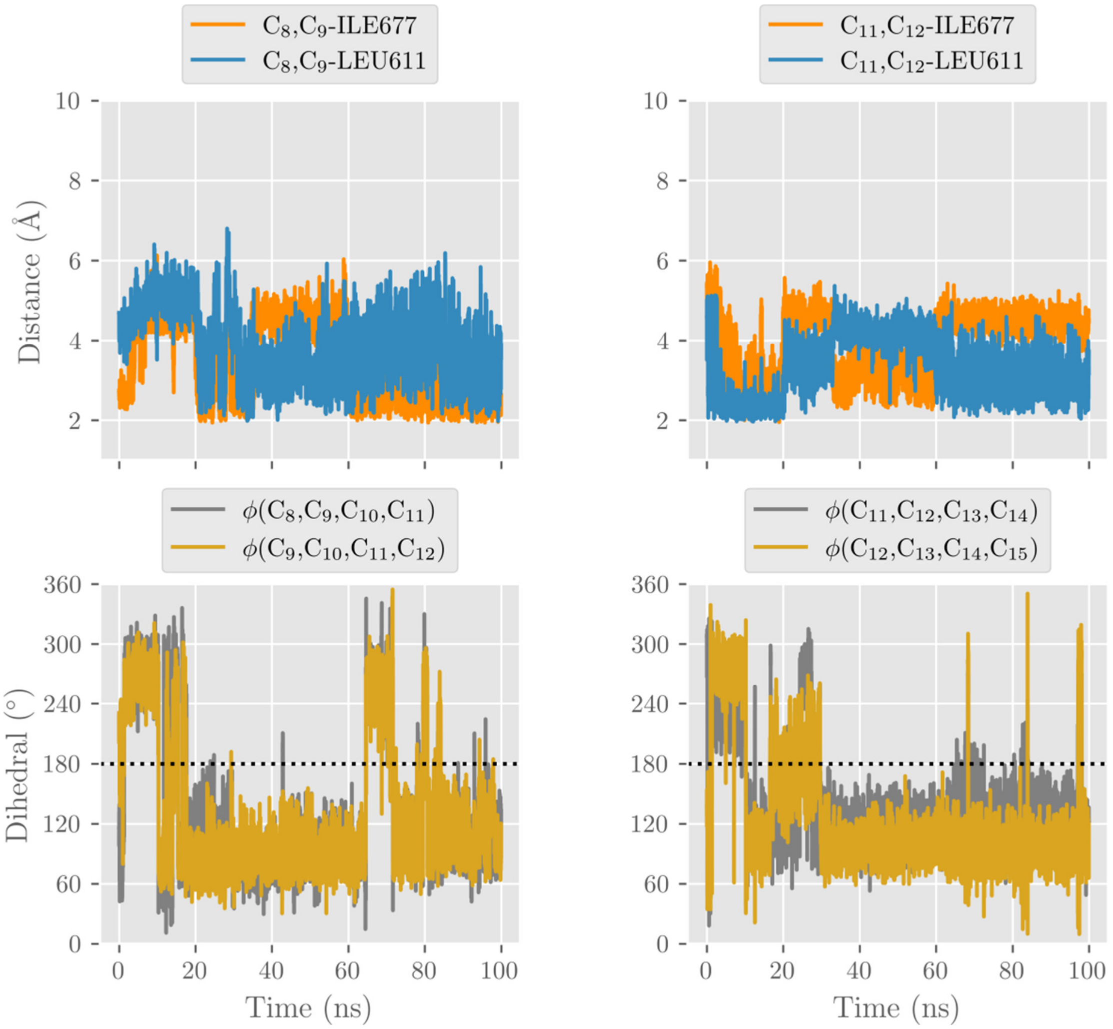

Figure S2. Molecular dynamics simulation of mouse Alox15b double mutant complexed with tail-first (tf) oriented arachidonic acid. For these simulations we first worked out 3D models for the Tyr603Asp+His604Val double mutant of mouse Alox15b. Then arachidonic acid (AA) was docked into the substrate binding pocket of the enzymes in its tail-first (tf) orientation, and the shortest distances (Å) from the C8-C9 and C11-C12 double bonds to terminal Ile677 and Leu611, and dihedral angles C8-C9-C10-C11, C9-C10-C11-C12, C11-C12-C13-C14 and C12-C13-C14-C15 along the Molecular Dynamics simulation were plotted. The C11-C12 (upper right) and C8-C9 (upper left) double bonds approach in a similar way to both residues, but with the result that the dihedral angles C11-C12-C13-C14 and C12-C13-C14-C15 (lower right) are closer to planarity (0° or 180°) than the dihedral angles C8-C9-C10-C11 and C9-C10-C11-C12 (lower left). That is, the abstraction of H10 in this case requires a broad motion of AA in the region of the C8-C9 double bond where the side chains of Ile677 and Leu611 significantly hinder the motion. Thus, H13 abstraction is preferred.

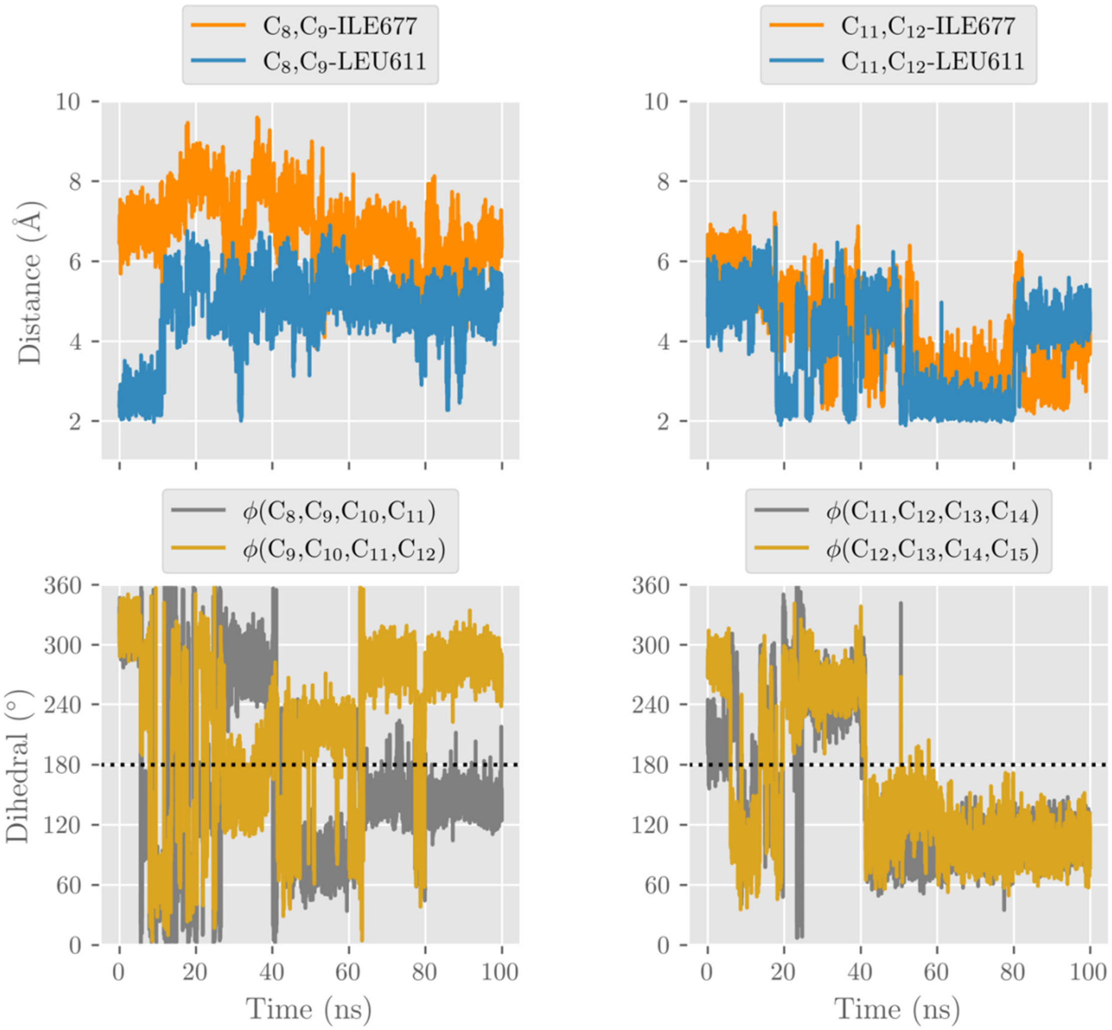

Figure S3. Molecular dynamics simulation of wild-type mouse alox15b complexed with head-first arachidonic acid. For these simulations we first worked out 3D models for the wild-type mouse alox15b. Then arachidonic acid (AA) was docked into the substrate binding pocket of the enzymes in its head-first (hf) orientation, and the shortest distances (Å) from the C8-C9 and C11-C12 double bonds to terminal Ile677 and Leu611, and dihedral angles C8-C9-C10-C11, C9-C10-C11-C12, C11-C12-C13-C14 and C12-C13-C14-C15 along the Molecular Dynamics simulation are plotted. C8-C9 distances to Ile677 and Leu611 (upper left) are long, meaning that the interaction between these groups is weak. In the case of C11-C12 (upper right), the interaction is stronger due to the shorter distances. Dihedral angles show that the double bonds around C10 (lower left) can move more freely, and they are closer to a planar situation (0 or 180º). The case of the double bonds around C12 (lower right) gives the opposite results: the dihedrals are closer to 90º and 270º, meaning that the double bonds are the farthest possible to a planar geometry. Thus, the steric hindrance favors the H10 abstraction.

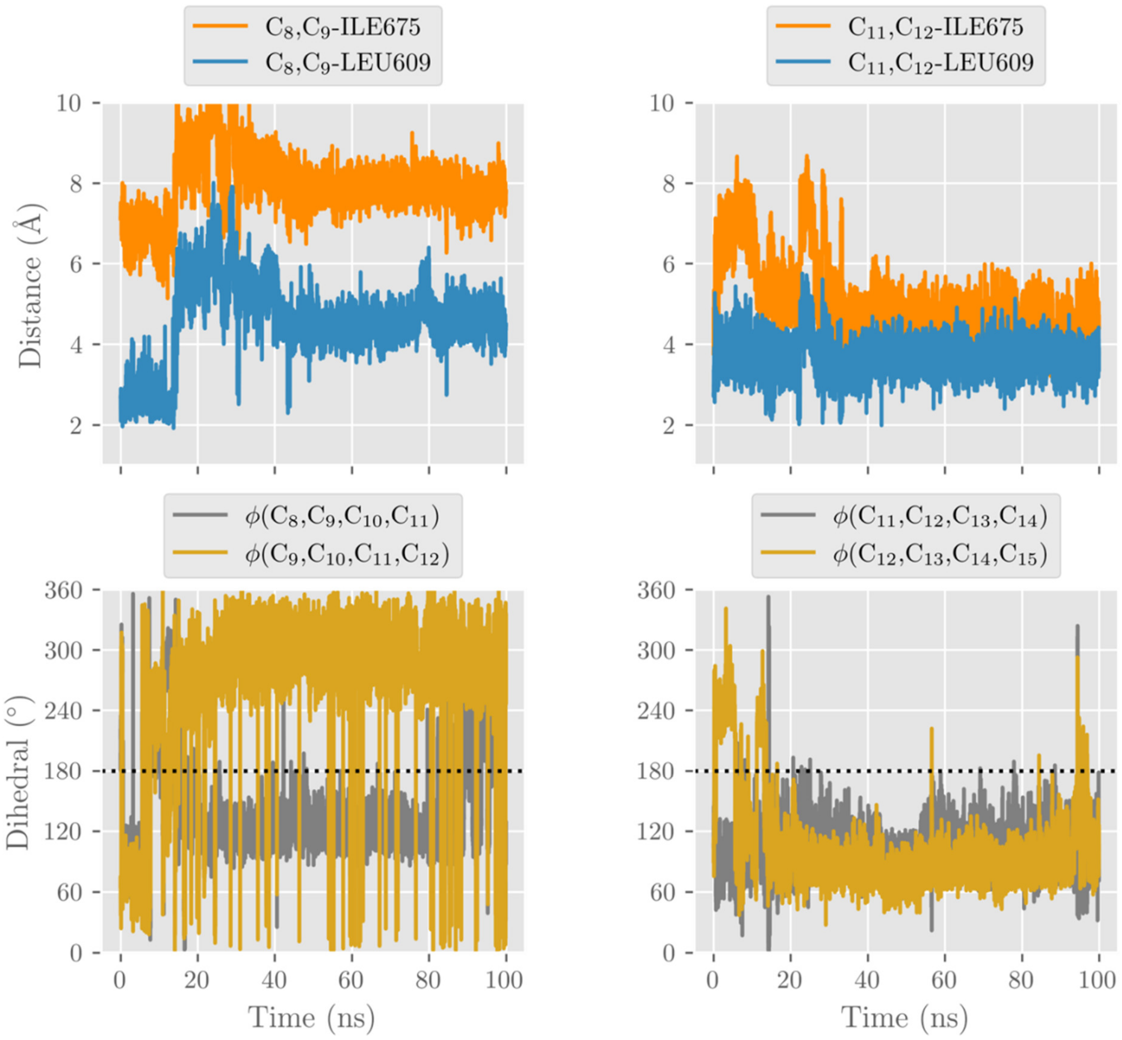

Figure S4. Molecular dynamics simulation of human ALOX15B double mutant complexed with head-first (hf) arachidonic acid. For these simulations we first worked out 3D models for the human ALOX15B double mutant. Then arachidonic acid (AA) was docked into the substrate binding pocket of the enzymes in its head-first (hf) orientation, and the shortest distances (Å) from the C8-C9 and C11-C12 double bonds to terminal Ile675 and Leu609, and dihedral angles C8-C9-C10-C11, C9-C10-C11-C12, C11-C12-C13-C14 and C12-C13-C14-C15 along the Molecular Dynamics simulation are plotted. C8-C9 interactions with Ile675 and Leu609 (upper left) are weak due to the long distances measured along the trajectory, while the same interactions with C11-C12 (upper right) double bond are stronger as their short distances reveal. As a consequence, the geometry around C10 is more planar as the dihedral angles C8-C9-C10-C11 and C9-C10-C11-C12 show (lower left). On the other hand, the stronger interaction with C11-C12 leads to a geometry around C13 farther from planarity, as the dihedrals C11-C12-C13-C14 and C12-C13-C14-C15 (lower right) show. Thus, achieving the planarity in the H-abstracted radical will be more favorable for the H10 abstraction than for the H13 abstraction.

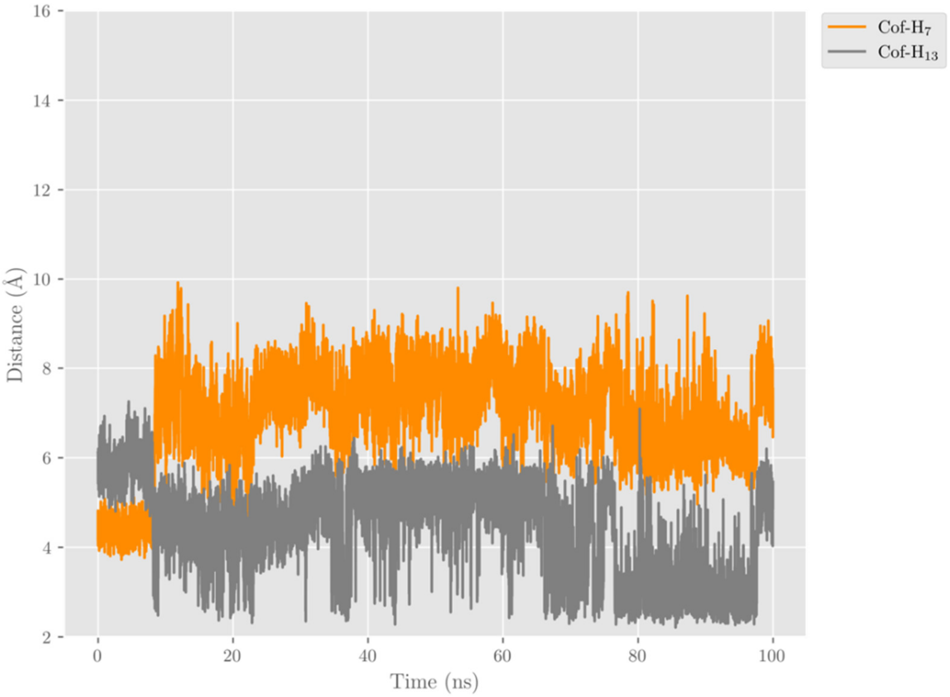

Figure S5. Molecular dynamics simulation of wildtype mouse Alox15b complexed with tail-first 8S-HETE. The distances from the oxygen atom in the Fe(III)-OH^- cofactor to the closest hydrogen atom attached to C7 (H7) and C13 (H13) along the Molecular Dynamics simulation are plotted. H13 is ready to be abstracted to yield the secondary 15-lipoxygenation, while H7 is far from the cofactor.
